# Breast Implant–Associated Epstein–Barr Virus‐Positive Diffuse Large B‐Cell Lymphoma

**DOI:** 10.1155/crh/7488322

**Published:** 2026-02-05

**Authors:** Thibault Maerten, Elsa Seijnhaeve, Wivine Bernard, Marc André, Gilles Crochet

**Affiliations:** ^1^ Hematology Department, CHU UCL Namur, Yvoir, 5530, Belgium; ^2^ Department of Pathology, Institute of Pathology and Genetics, Gosselies, 6041, Belgium, ipg.be

**Keywords:** BIA-DLBCL, breast implant-associated diffuse large B-cell lymphoma, diffuse large B-cell lymphoma associated with chronic inflammation, DLBCL-CI, Epstein–Barr virus, FA-LBCL, fibrin-associated large

## Abstract

Breast implant–associated diffuse large B‐cell lymphoma (BIA‐DLBCL) is an extremely rare entity, often misdiagnosed as breast implant–associated anaplastic large cell lymphoma (BIA‐ALCL). Unlike BIA‐ALCL, which is a T‐cell neoplasm, BIA‐DLBCL shows B‐cell immunophenotype and is frequently associated with Epstein–Barr virus (EBV). Few cases have been reported and its optimal management remains unclear. We report the case of a 45‐year‐old woman with a history of breast augmentation surgery using textured silicone implants. She presented with left breast pain and deformity. Histopathological examination of the periprosthetic capsule revealed large atypical lymphoid cells, expressing CD20, CD19, PAX5, CD79a, and CD30, with EBV RNA positivity and absence of T‐cell markers. There was no capsular rupture. PET‐CT scanning showed hypermetabolic activity around the implant and ipsilateral axillary lymphadenopathy, without systemic involvement. A diagnosis of BIA‐DLBCL was retained. The patient underwent total capsulectomy without adjuvant therapy. At 30‐month follow‐up, she remains in complete clinical and radiological remission. BIA‐DLBCL is an increasingly reported entity which in most cases can be classified within the spectrum of fibrin‐associated large B‐cell lymphoma (FA‐LBCL). While surgical excision alone may be sufficient for localized disease, the rarity of this lymphoma highlights the urgent need for more comprehensive data, particularly long‐term survival outcomes, to refine classification and therapeutic recommendations.

## 1. Introduction

Breast implant–associated diffuse large B‐cell lymphoma (BIA‐DLBCL) is a rare entity characterized by a clonal proliferation of B‐cells in the context of a breast prosthesis. It can be easily confused with breast implant–associated anaplastic large cell lymphoma (BIA‐ALCL), a much more common and well‐recognized T‐cell lymphoma. In both conditions, large, atypical cells with strong CD30 expression may be observed. However, BIA‐DLBCL typically expresses B‐cell lineage markers, such as CD20, PAX5, and CD79a, and is most often classified as a nongerminal center B‐cell subtype according to the Hans algorithm. Additionally, there is a notable association with Epstein–Barr virus (EBV), frequently demonstrated by EBV‐encoded RNA (EBER) in situ hybridization. T‐cell markers, including CD2, CD3, CD5, and CD8, are typically negative [1].

The exact incidence of BIA‐DLBCL is difficult to determine. A recent study involving 56,000 women who underwent mastectomy with implant‐based reconstruction, with a median follow‐up of 83 months, reported four cases of BIA‐DLBCL, corresponding to an estimated incidence of 9 cases per million person‐years [2]. By comparison, the incidence of BIA‐ALCL is also imprecise due to under‐reporting and incomplete registries but is estimated to range from 1 in 1000 to 1 in 10,000 for textured implants, making it significantly more common [3]. BIA‐DLBCL must also be distinguished from diffuse large B‐cell lymphoma not otherwise specified (DLBCL, NOS) involving the breast in the absence of an implant‐related context [4].

Regarding classification, BIA‐ALCL has been recognized as a distinct entity in both the 2022 World Health Organization (WHO) Classification and the 2022 International Consensus Classification (ICC) of hematologic malignancies [5, 6]. In contrast, BIA‐DLBCL does not yet have its own dedicated classification and is generally considered either as a diffuse large B‐cell lymphoma associated with chronic inflammation (DLBCL‐CI) or a fibrin‐associated large B‐cell lymphoma (FA‐LBCL) [7]. These entities are now well separated in both WHO and ICC classifications [5, 6].

Based on clinical and histopathological characteristics, most cases of BIA‐DLBCL appear to align with the features of FA‐LBCL [1, 7]. The prognosis is generally favorable, and surgical resection alone is often sufficient. However, due to the rarity of this condition, standardized treatment guidelines have not yet been established. Here, we report a new case of BIA‐DLBCL recently diagnosed in our center.

## 2. Case Report

A 45‐year‐old woman underwent cosmetic breast augmentation in 2017 with the placement of 330 g round, textured, silicone breast implants (TSM type) in the prepectoral position combined with mastopexy. Her past medical history included obesity managed with semaglutide and asthma. She had no other significant comorbidities. In July 2022, she began experiencing pain in the left breast. A mammogram at that time was unremarkable. Due to persistent pain and progressive breast deformity, she underwent surgical intervention on December 7, 2022, consisting of capsulotomy and implant repositioning. Histopathological analysis of the periprosthetic capsule revealed large CD20+ lymphoid cells with prominent nucleoli suspicious for diffuse large B‐cell lymphoma. A staging PET‐CT scan showed moderate hypermetabolic activity surrounding the left breast implant and ipsilateral axillary lymphadenopathy, with no evidence of distant disease (Figure 1). The patient reported night sweats over the previous month, without fever or weight loss. Laboratory tests were within normal limits.

Figure 1PET‐CT (a, b) showing moderate hypermetabolic activity surrounding the left breast implant and ipsilateral axillary lymphadenopathy.

**Figure:**
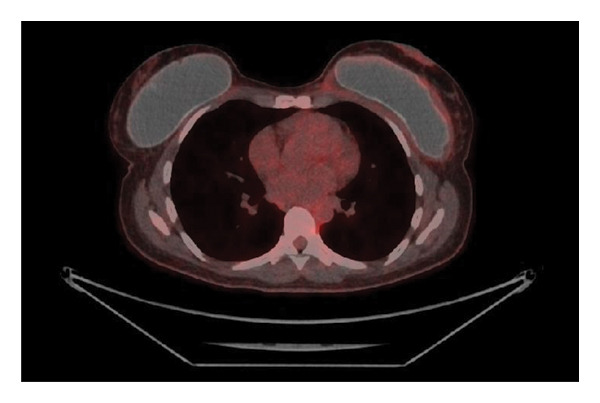
(a)

**Figure:**
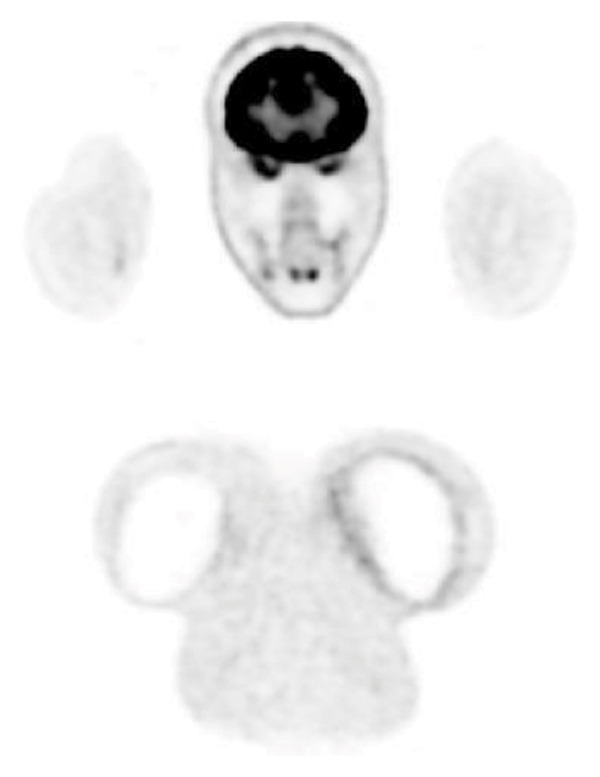
(b)

A second surgical procedure was performed on January 10, 2023, consisting of a total bilateral capsulectomy. Histopathological examination of the left implant showed a fibrous capsule lined with fibrin deposits. Within the fibrin, an embedded cluster of atypical large lymphoid cells was found. These cells were confined to the inner surface of the periprosthetic capsule without capsular rupture. B‐cell markers were strongly expressed (CD20, CD19, PAX5, and CD79a), and CD30 and EBER were positive (Figure 2). T‐cell markers (CD3, CD4, CD8, CD2, CD5, CD7, and ALK) were negative. Based on these findings, a diagnosis of BIA‐DLBCL was established. The right implant showed no signs of malignancy.

Figure 2Histopathological findings: (a) Hematoxylin and eosin (H&E) staining of the capsule showing clusters of large atypical lymphoid cells with prominent nucleoli, consistent with diffuse large B‐cell lymphoma. (b) EBER in situ hybridization highlights EBV‐positive large B‐cells (black nuclear signals) embedded in the fibrous capsule. (c) Absence of membrane expression of CD5. The blue nuclei represent counterstaining.

**Figure:**
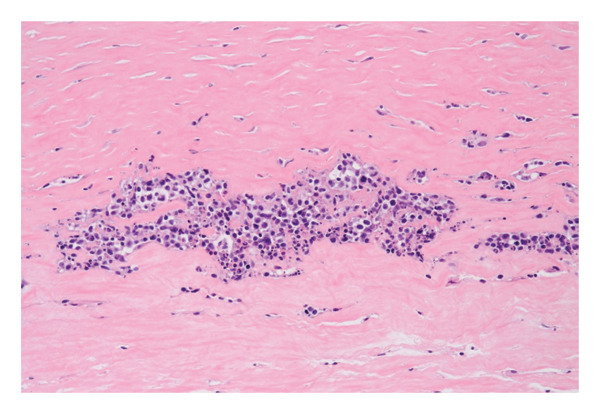
(a)

**Figure:**
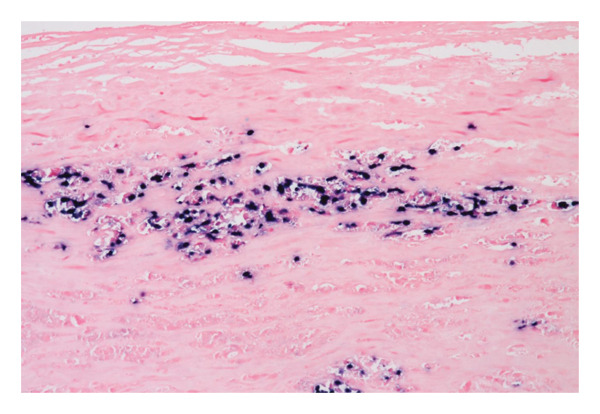
(b)

**Figure:**
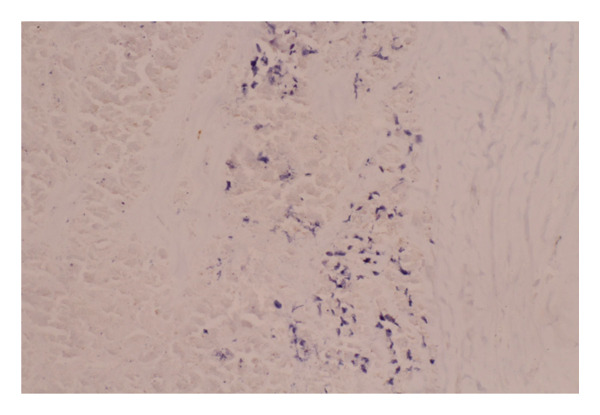
(c)

A follow‐up PET‐CT scan on March 3, 2023, showed complete metabolic response. The patient also reported complete resolution of night sweats. Given the localized nature of the disease and absence of systemic involvement, a watchful waiting strategy was adopted without adjuvant therapy. No new implants were subsequently placed on either side. As of August 2025, 30 months of posttreatment, the patient remains in complete clinical and radiological remission.

## 3. Discussion

The review by Vets et al. provides a comprehensive synthesis of 21 reported cases of BIA‐DLBCL [1], incorporating previously published cases [7–11]. Since their review, four additional cases have been reported [12–15]. Including our current case, the total number of documented BIA‐DLBCL cases now reaches 26.

Whether BIA‐DLBCL belongs to FA‐LBCL or DLBCL‐CI is still a matter of debate. DLBCL‐CI is associated with long‐standing localized inflammation, often in response to foreign materials, such as metallic implants, chronic osteomyelitis, or surgical mesh, and shows a strong association with EBV [7]. DLBCL‐CI is usually considered an aggressive lymphoma with a poor prognosis unlike most reported cases of BIA‐DLBCL [1].

FA‐LBCL by comparison is characterized by the absence of a true mass and involves lymphoma cells embedded in fibrinous or necrotic material, often found incidentally in chronic hematomas or around prosthetic cardiac valves [8]. This variant is also EBV‐positive but typically follows an indolent course with favorable outcomes and minimal clinical symptoms. Based on these features, our case is most consistent with the diagnosis of FA‐LBCL, in line with the majority of published reports.

Currently, there are no standardized staging criteria for BIA‐DLBCL. In clinical practice, it appears reasonable to use the TNM classification developed for BIA‐ALCL [16]. In our case, the disease was staged as pT3N0M0.

Treatment strategies in the literature have largely been extrapolated from BIA‐ALCL guidelines: surgical capsulectomy alone for localized disease and immunochemotherapy, typically R‐CHOP, for more extensive involvement. This surgical approach alone was used in 21 of the 26 reported cases, all presenting with localized disease. Five patients (19%) received adjuvant immunochemotherapy after surgery. Among these, one had axillary lymph node involvement, and another was staged as pT4N0M0. The remaining three patients also had localized disease but received systemic therapy based on local institutional decisions. In our case, we elected to perform capsulectomy alone without adjuvant chemotherapy given the localized nature of the disease and the absence of capsular rupture. This approach appears consistent with the limited evidence available in the literature.

Short‐term outcomes were favorable in patients treated with surgery alone. However, long‐term data remain scarce. Continued reporting of BIA‐DLBCL cases is essential to deepen our understanding and refine therapeutic approaches. The accumulation of well‐documented cases, with extended follow‐up, will be necessary to develop evidence‐based, standardized, treatment guidelines.

## 4. Conclusion

We report another case of BIA‐DLBCL. BIA‐DLBCL is an increasingly recognized entity that, in most cases, appears to fall within the spectrum of FA‐LBCL. While outcomes are generally favorable with surgery alone, long‐term survival data are still lacking and remain essential to guide evidence‐based therapeutic recommendations.

**Nomenclature**
BIA‐ALCL: Breast implant–associated anaplastic large cell lymphoma
BIA‐DLBCL: Breast implant–associated diffuse large B‐cell lymphoma
DLBCL‐CI: Diffuse large B‐cell lymphoma with chronic inflammation
CT: Computed tomography
DLBCL, NOS: Diffuse large B‐cell lymphoma not otherwise specified
EBER: Epstein–Barr virus–encoded RNA
EBV: Epstein–Barr virus
FA‐LBCL: Fibrin‐associated large B‐cell lymphoma
ICC: International Consensus Classification
PET: Positron emission tomography
R‐CHOP: Rituximab, cyclophosphamide, doxorubicin hydrochloride, vincristine, and prednisone
WHO: World Health Organization

## Author Contributions

Thibault Maerten: data acquisition, interpretation of the results, and writing of the manuscript; Elsa Seijnhaeve contributed perspective as a pathologist and the histological images; Gilles Crochet, Wivine Bernard, and Marc André supervised the manuscript writing and verified the data.

## Funding

The study is funded by the authors’ own resources.

## Disclosure

All authors have read and agreed to the published version of this manuscript.

## Consent

The patient gave full and informed consent for publication of deidentified information in this case.

## Conflicts of Interest

Gilles Crochet has received consultancy and conference grants from Roche, Kite/Gilead, BMS, and Abbvie. The other authors declare no conflicts of interest.

## Data Availability

The data supporting this case study are available from the corresponding author upon request.
